# Risk Assessment of Nitrite and Nitrate Intake from Processed Meat Products: Results from the Hellenic National Nutrition and Health Survey (HNNHS)

**DOI:** 10.3390/ijerph191912800

**Published:** 2022-10-06

**Authors:** Sotiria Kotopoulou, Antonis Zampelas, Emmanuella Magriplis

**Affiliations:** 1 Department of Food Science and Human Nutrition, Agricultural University of Athens, Iera Odos 75, 11855 Athens, Greece; 2 Hellenic Food Authority, Leoforos Kifissias 124 & Iatridou 2, 11526 Athens, Greece

**Keywords:** nitric compounds, food additives, MPLs, dietary exposure, dietary intake, risk, meat consumption, processing, food group contribution, Greece

## Abstract

Long-term exposure to a high nitrite and nitrate intake through processed meat is of concern, as it has been related to adverse health effects. Individual consumption data from 2152 participants (46.7% males) in the Hellenic National Nutrition and Health Survey (HNNHS) were linked with current Maximum Permitted Levels (MPLs) to calculate exposure to nitrite and nitrate from processed meat products (assessed as nitrite equivalent), evaluate potential risk and identify the major contributors. Processed meat intakes were determined by combining data from 24 h recalls and frequency of consumption reported in Food Propensity Questionnaires (FPQs). Median exposure was estimated to be within safe levels for all population groups. However, 6.6% (*n* = 143) of the consumers exceeded the Acceptable Daily Intake (ADI) of nitrite (0.07 mg/kg bw/day), of which 20.3% were children aged 0–9 years (N = 29) (15.3% of all children participants in the study, N = 190). In total, pork meat was the major contributor (41.5%), followed by turkey meat (32.7%) and sausages (23.8%), although contribution variations were found among age groups. The outcomes are of public health concern, especially exposure among children, and future research is warranted to evaluate possible associations with health effects, by using more refined occurrence data if available.

## 1. Introduction

Processed meat consumption has been linked with human carcinogenesis [1], based primarily on epidemiologic studies evaluating risk factors of colorectal cancer (CRC). Recommendations from the World Health Organization (WHO) [2], implemented also in Greece [3], suggest limiting or avoiding consumption, since no safe intake has been established. Specifically, processed meat is any meat that has been flavored or preserved by salting, curing, fermenting, smoking, or other techniques [4]. It may contain a high concentration of nitrite (potassium nitrite-E249 and sodium nitrite-E250) and nitrate (sodium nitrate-E251 and potassium nitrate-E252), both of which are legally and widely used food additives in the European Union [5], to fix color, limit microbial growth (particularly of Clostridium botulinum), or develop a distinguishable flavor [6]. However, controversies regarding the healthiness of nitrite and nitrate have been reported and their consumption should therefore be separately monitored from red meat. The hazard of dietary nitrite and nitrate for human health has previously been thoroughly reviewed [7] and an increased risk of cancers, especially of the gastrointestinal tract, when ingested from processed meat has been suggested. This is principally attributable to the production of genotoxic N-nitroso compounds (NOCs), nitrosamines, and nitrosamides under the proper conditions (pH, reactant concentration), as well as the lack of nitrosation inhibitors such as vitamin C [1]. Legislative Maximum Permitted Levels (MPLs) for the use of nitrite and nitrate in processed meat products have been set [5] to ensure that the desired effect of the product is obtained without exceeding safe levels for human health. 

Nutrition transition, linked with lifestyle changes seen in recent decades, has been characterized by a decrease in plant foods consumption and an increase in animal-based products consumption [8], as well as an increased consumption of processed and packaged foods [9]. This trend, therefore, has increased food additive consumption, including nitrite and nitrate, raising the risk of exceeding Acceptable Daily Intake levels (ADIs) [10]. ADI is a health-based reference value that characterizes the hazard and is used to represent the safe levels of the quantity of additives in food or drinking water that may be ingested every day during a lifetime, without posing significant harm to health [11]. The ADI for nitrite is 0.07 mg nitrite ion/kg bw/day [12] and for nitrate it is 3.7 mg nitrate ion/kg bw/day [13]. The toxicity of a food additive and its dietary intake determine the extent to which it may represent a health concern [9] and negative health impacts may arise when ADIs are surpassed [14]. Thus, intakes must be monitored by member states in order to facilitate risk assessment [5], through a scientifically based four-step procedure comprising of hazard identification, hazard characterization, exposure assessment and risk characterization, which is used to estimate the proportion of individuals and age-groups that exceed these limits, for public health monitoring [15].

Several studies have been published to assess dietary exposure to nitrite and nitrate from processed meat products in other countries [16,17,18,19,20,21,22,23,24]; however, to our knowledge, no relevant research has been conducted to date for Greek consumers. Moreover, Greeks seem to follow a dietary pattern that differs significantly from the Mediterranean diet, presenting a high intake of red meat and fast food [25] and the gradual adoption of harmful eating habits appears to have accelerated since the onset of the Greek debt crisis [26]. Consequently, the objectives of this study were as follows: (a) estimate the daily nitrite and nitrate intake from processed meat products consumption; (b) assess the potential risk of exceeding the ADI; and (c) identify the major contributors, all in total and by age group, using a nationally representative sample.

## 2. Materials and Methods

### 2.1. Study Design and Subjects

Participants from the Hellenic National Nutrition and Health Survey (HNNHS), a nationally representative health study of the general population conducted from September 2013 to May 2015, were included in the study; pregnant or nursing women, institutionalized individuals and those serving in the military forces were excluded. Multistage stratified sampling was based on data from the latest Hellenic Statistical Authority’s geographical density criteria by area, age, and sex. Trained professionals interviewed all research participants using Computer Assisted Personal Interview (CAPI) software to collect data on socio-demographics parameters, dietary intake, and lifestyle. Details of the survey, as well as of the questionnaires used, have been published elsewhere [27,28] The Hellenic Data Protection Authority (HDPA) and the Ethics Committee of the Department of Food Science and Human Nutrition of the Agricultural University of Athens approved the activities after receiving individual permission and approval.

### 2.2. Exposure Assessment

#### 2.2.1. Food Consumption Data

- Participants

Data of 2152 (47.5% of HNNHS participants, 46.7% males) individuals across all age groups consuming processed meat products for which the addition of nitrite and nitrate is permitted [5] (henceforth: consumers) were retrieved to be included in our risk assessment, out of a total of 4532 participants in the HNNHS (henceforth: general population). The population enrolled was primarily classified in age groups following the clustering suggested by EFSA [29]. For better power of statistical analysis, infants (<11 months, *n* = 1) and toddlers (<3 years, *n* = 14) were moved to the upper class of other children (3–9 years) and were all grouped as children (0–9 years), and individuals ≥65 years old were not further divided as elderly (65–74 years) and very elderly (≥75 years). Even after further subgrouping of adults aged 18–64 years, the size of all age groups was large enough for the calculation of the 95th percentile of exposure (N ≥ 60) [29]. The age classification finally adopted included children: 0–9 years, adolescents: 10–17 years, young adults: 18–30 years, adults: 31–50 years, older adults: 51–64 years and elderly: ≥65 years. Misreporters, defined as individuals reporting energy intake <500 and >6000 kcal/day (N = 15, 3 under-reporters and 12 over-reporters) were also included, as recommended by EFSA to always use a conservative approach [30,31].

- Processed meat consumption frequency

For 1890 (87.8%) participants, the dietary intakes were collected by two non-sequential 24 h recalls. The 24 h recall techniques have been described in detail elsewhere [27]. The remaining 12.2% of participants (N = 262) took part in the dietary survey for only one day. Individuals that supply data for one dietary recall are typically excluded from chronic exposure assessments, since at least two survey days per subject are normally required [29]. To overcome this and adjust for the quantity of the processed meat consumed over time, the frequency of consumption of processed meat or meat containing dishes during a 24 h recall, as provided in validated Food Propensity Questionnaires (FPQs), was used. “Νever”, “less than once a month”, “1–3 times per month”, “once a week”, “2–4 times a week”, “5–6 times a week”, “every day”, “2–3 times a day”, “4–5 times a day”, “5–6 times a day” were among the options in the FPQs. Thus, all consumers were included and any processed meat consumption, whether whole or in mixed dishes/recipes, was evaluated. The frequency of consumption was converted to servings per day by dividing the mean of the stated frequencies by the overall number of days (for instance, for a frequency of 1–3 times per month, the mean was 2 times per month and was divided by 30 to result in 0.067 servings per day). To acquire a relative intake over time and reduce variability, this value was multiplied by all participants’ individual processed meat-eating events (in grams). Individuals who replied “never” in the FPQs but had records of processed meat consumption were additionally assigned to appropriate consumption frequencies.

#### 2.2.2. Occurrence Data

- Food groups classification

Processed meat products in which the use of nitrite and nitrate is authorized [5], as well as dishes/recipes containing those, were selected from HNNHS. Composite dishes/recipes were broken down to their components and then all products were classified in processed meat categories according to food classification and description system FoodEx2 [32], in order to consequently attribute the legislative MPLs and identify the major contributors.

Overall, 17 FoodEx2 food groups were considered in the present exposure assessment, based on their basic term codes. They were subsequently summed up into 4 broader food groups, including subcategories where needed, such as pork meat (bacon, ham, other), poultry meat (chicken, turkey), sausages and meat specialties. Pork and poultry meat together fall under the broader FoodEx category of preserved meat. The unique foods and composite dishes/recipes from HNNHS considered are listed in Table S1 of the Supplementary Materials per food group. Processed meats were more frequently consumed as part of mixed dishes and recipes (75.1%), mainly in toasts/sandwiches (44.2%) and pizzas (14.2%), as can be seen in Table S2. 

The European Commission and EFSA suggest using a stepwise procedure to estimate additive intakes and among the recommended Tiers; the second one, known as the regulatory maximum level exposure assessment scenario (Tier 2), was used in this study since it links actual national food consumption data and MPLs [33,34].

- Maximum Permitted Limits (MPLs)

Nitrite and nitrate were assumed to be present in processed meat products at a concentration level equal to their legislative MPLs [5]. To attribute MPLs, FoodEx2 categories were matched with food categories of Regulation (EC) 1333/2008, following the mapping conducted by EFSA [35]. The food groups with their corresponding MPLs used in our assessment are listed in Table S3 of the Supplementary Materials.

- Estimated nitrite and nitrate intake

Individual processed meat intakes per FoodEx2 category, as adjusted before with frequencies of consumption and after being converted in kg, were multiplied with corresponding MPLs (mg/kg), resulting in nitrite and/or nitrate intakes (in mg) per individual and eating event. The total nitrite and nitrate intake per participant were determined, averaged in case of two days of recall, and divided by individual body weight in order to acquire the quantified dietary exposure of nitrite and nitrate, separately, for each subject in the study in mg/kg bw/day [13]. 

Some aspects about dietary nitrate exposure from processed meat products were further considered. The nitrate intake when used as a food additive was estimated by EFSA, using the refined scenario, and it was found to be less than 5% of the overall dietary nitrate intake [13]. Generally, the nitrate intake is much higher from vegetables and vegetable products than from additive sources [36]; thus, the in vivo converted amount is hardly taken into consideration when dietary exposure to nitrite and nitrate through processed foods is studied [20]. Furthermore, the toxicity of nitrate is determined by its conversion to nitrite and potential endogenous nitrosation and only when nitrate is ingested at its ADI level of 3.7 mg/kg bw/day, the in vivo nitrate-to-nitrite conversion in the human body may result in an exposure of significantly above the ADI for nitrite [1]. Finally, the nitrate-to-nitrite conversion factor uncertainty has been discussed previously [37].

The highly conservative Tier 2 approach used in our study revealed a null median nitrate intake from processed meat products among Greeks (Table S4), both in the general population and in consumers. Even at the 95th percentile, the intake was 0.003 mg/kg bw/day for the general population and 0.012 mg/kg bw/day for consumers, which is well below the ADI of 3.7 mg/kg bw/day, suggesting that there is no risk for surpassing the ADI of nitrite from the in vivo conversion of nitrate-to-nitrite in the human body. Despite the low dietary nitrate exposure estimated, its conversion to nitrite (in mg) was further considered in order to account for the co-exposure of nitrite and nitrate intake, by exploring the following three different conversion factors: 1% and 9% suggested by EFSA as the lower and higher nitrate-to-nitrite conversion in the body [13] and 2.3% as a median conversion factor used in research before [37]. The nitrate-to-nitrite conversion (mg) was then added to the direct intake of nitrite and total nitrite intake was assessed for each subject by dividing by individual body weight. 

As expected, differences between estimates were negligible (Table S4). Additionally, the highest conversion factor of 9% better serves the worst case scenario, already adopted with the maximum regulatory exposure assessment scenario used to assess exposure. Thus, the co-exposure of daily intake of nitrite and nitrate from processed meat products as determined by applying the 9% nitrate-to-nitrite conversion factor—hereafter also referred to as total nitrite intake—was further used to analyze, present, and discuss data in this study.

- Estimated food groups contribution to daily total nitrite intake

To calculate how much each food group contributes to the daily total nitrite exposure from processed meats, the following formula was used: (Total exposure per food group per day/Total exposure per day) × 100. This contribution was also estimated per age group in total (Total exposure per food group per day per age group/Total exposure per day) × 100 and among consumers of the same age group: (Total exposure per food group per day per age group/Total exposure per day in the reference age group) × 100.

### 2.3. Risk Characterization

The total nitrite intake exposure levels estimated were compared with the ADI value of nitrite (0.07 mg/kg bw/day), to identify the subgroups or extreme consumers that might be at risk of exceeding the ADI and which may have adverse impacts on their health. Dietary nitrite exposures were also reported as a percentage contribution to ADI [12], and if the percentage was less than 100% it was concluded that there was no risk from exposure to the additive.

### 2.4. Other Parameters

Trained health professionals interviewed participants to obtain sociodemographic and anthropometric data, gathering information on age, gender, and educational level. The educational level was divided into the following three categories: up to 6 years of schooling, 12 years of schooling and higher education (including colleges). Smoking patterns and levels of physical activity were also evaluated. Individuals were categorized as ex-smokers if they had been at least 30 days smoke free, smokers, or never-smokers. Physical activity (PA) was defined according to the International Physical Activity Questionnaire (IPAQ), as per the calculation guidelines [38]. Sedentary status was assigned to people who scored below the light activity level. Body Mass Index (BMI) was calculated using the measurements of weight (kg) and height (m), using the following formula: weight/height^2^ (kg/m^2^). Weight status was categorized as healthy weight ≤ 25 kg/m^2^, 25 ≤ overweight < 30 kg/m^2^, and obese ≥ 30 kg/m^2^. Children and adolescents were classified using the extended International Obesity Task Force (IOTF) tables [39]. Total fat, trans fatty acid (TFA) and saturated fat acid (SFA) intakes had been estimated before, as % of total energy intakes [40]. Sodium intake was categorized per approximately 800 mg intake as <1500, ≥1500 and <2300 and ≥2300. Adherence to the Mediterranean diet was evaluated using the MedDiet score, ranging from 0 to 55 [41]. The variable was dichotomized to two final MedDiet categories, <23 and ≥23 for low and high adherence, respectively, based on the median value of the population, since it has been shown that for every 11-unit rise in Med Diet score, there was a 37% odds decrease in acute coronary event [42]. 

### 2.5. Statistical Analysis

Baseline variables were stratified based on participants’ level of total nitrite intake, of below or above the ADI of 0.07 mg/kg bw/day of nitrite, to assess significant differences. Continuous variables were presented as mean (standard deviation-sd) when normally distributed and as median (Interquartile Range-IQR: 25th percentile, 75th percentile) for skewed distributions. Categorical variables were presented as frequencies. The nonparametric Kruskall–Wallis rank sum and ANOVA test were employed to test group differences for skewed and continuous variables, respectively. For categorical variables, chi square testing was conducted. A *p*-value < 0.05 was considered statistically significant. Where significant differences were identified, the variables were entered into a logistic regression model to account for potential confounding. Variables considered in the logistic regression were age group, sex, weight, employment status, total energy intake, sodium intake category and MedDiet category. Missing age, weight and frequency of consumption data were imputed. All data statistical analyses were carried out using the STATA 13.0 (StataCorp, College Station, TX, USA) statistical software.

## 3. Results

Median total nitrite intake from processed meat products was found to be 0.007 (0.003, 0.02) mg/kg bw/day, accounting for 10% (4.3%, 28.6%) of the ADI of 0.07 mg/kg bw/day for nitrite. Table S5 presents the distribution of daily nitrite intake in mg/kg bw/day and as a percentage of the ADI, in total and per age group and sex for consumers only, which ranged from 0.003 (0.001, 0.007) mg/kg bw/day in the elderly (≥6 ears) to 0.02 (0.008, 0.042) mg/kg bw/day in children (0–9 years). The results are summarized in Table 1.

**Figure 1:**
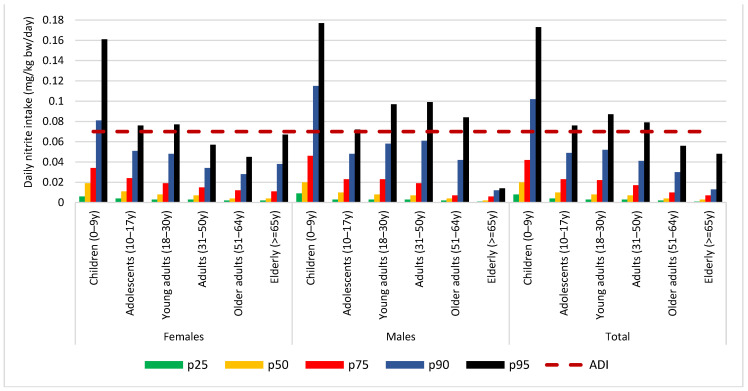
Distribution of daily total nitrite intake (mg/kg bw/day) by sex and age group, in comparison to ADI of 0.07 mg/kg bw/day for nitrite.

Figure 1 shows that ADI was surpassed in most of the age groups and in total (118.6% of the ADI), when daily intake was estimated at the 95th percentile. Specifically, all females up to 30 years and all males up to 64 years old surpassed it. Additionally, nitrite exposure estimated for children 0–9 years exceeded the ADI already at the 90th percentile for both sexes (115.7% of the ADI for girls and 164.3% of the ADI for boys).

The main characteristics and baseline variables differences stratified by level of intake (above and below the ADI of 0.07 mg/kg bw/day) of the population studied are summarized in Table 2. A total of 6.6% (N = 143) of consumers exceeded the ADI of nitrite from the ingestion of processed meat products only (3.2% of the general population). When over consumers (total energy > 6000 kcal/day, N = 12) were excluded, the proportion of those exceeding the ADI was estimated to be 6.5% (N = 138), hence the results did not differ. Among those exceeding the ADI, young adults aged 18–30 years had the highest proportion (37.1%), followed by adults aged 31–50 years (28%) and children aged 0–9 years (20.3%). Overall, more males exceeded the ADI (60.8%) than females (39.2%). Significant differences were found in age and age groups, sex, weight, employment status, total energy intake, total sodium intake (in both mg and category) and in MedDiet score and category. The area of residence, marital status, education level, smoking status, physical activity level and total fat, TFA and SFA intakes, and BMI/weight status of children only did not significantly differ. The BMI of adults significantly differed by ADI levels of intake, but this was not significant by weight status.

A logistic regression model was used to assess the likelihood of consuming total nitrite from processed meats above the ADI (Table S6). The results revealed that high adherence to Mediterranean diet (MedDiet score ≥ 23) significantly decreased the odds of exceeding the ADI of nitrite from the consumption of processed meat products (OR: 0.6, 95% CI: 0.36–0.90). Among the consumers of processed meat products in our study, 54.3% reported a frequency of consumption of once a week and 22.9% reported a frequency of 1 to 3 times per month. Among those with an intake of total nitrite exceeding the ADI, however, 39.2% consumed processed meat products 2 to 4 times a week, 19.6% consumed them every day, 16.8% from 5 to 6 times a week and a 12.6% from 2 to 3 times a day (Table S7). 

**Figure 2:**
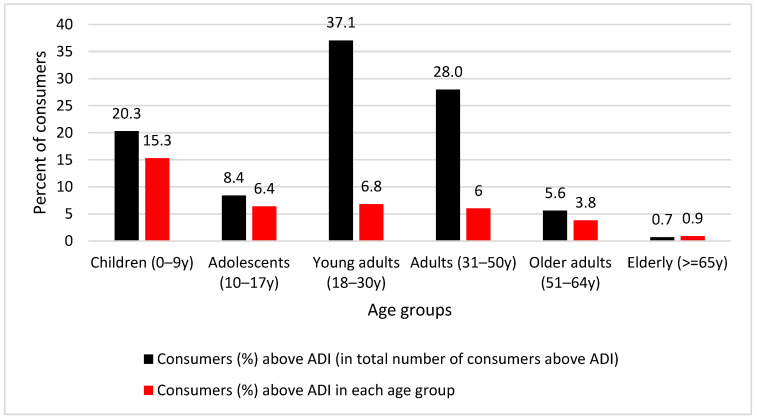
Proportion of the population per age group that exceeds the ADI of 0.07 mg/kg bw/day for nitrite in total exceeders and within the same age group.

Figure 2 depicts individuals (%) that exceeded the ADI per sex and age group over total number of exceeders (N = 143) as well as over the total number of participants of the same age group (the data are presented in detail in Table S8). Specifically, 15.3% (N = 29) of children aged 0–9 years that participated in the study (N = 190) were found to exceed the ADI, with a higher proportion of males over females (18.1% and 11.8%, respectively). 

**Figure 3:**
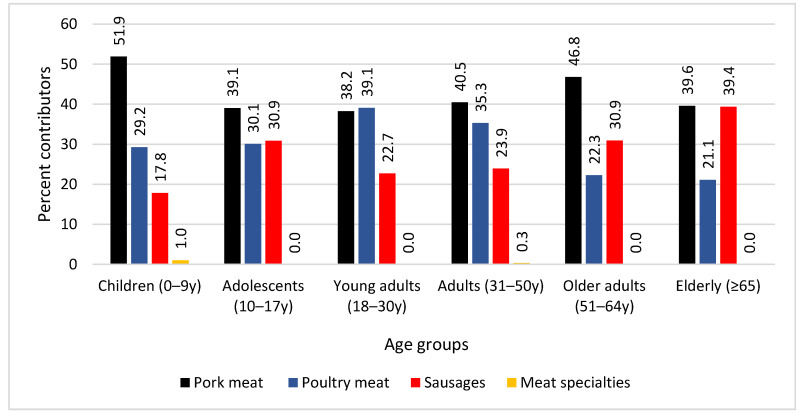
Main processed meat food groups contributing to total nitrite intake among consumers of the same age group.

The major contributors to total nitrite exposure, when based on FoodEx2 groups classification, was by far A023T-Cooked Turkey Meat (26.83%), followed by A0EYP-Preserved or partly preserved sausages (18.23%), A023H-Cooked cured (or seasoned) pork meat (17.56%) and A022T-Ham pork (13.04%) (Figure S1 in the Supplementary Materials). When FoodEx2 groups were divided into broader food groups, as already presented in Table S1, products from pork meat in general, including ham, bacon and other pork meats, contributed the most to nitrite exposure (41.53%), in relation to poultry meat products (34.39%), sausages (23.82%) and meat specialties (0.26%) (Table S9). Nitrite exposure from poultry meat was primarily due to turkey meat (32.7% of the total 34.4%), whereas exposure from pork meat was primarily due to non-specific products (20.4%) and ham (17.7%). Figure 3 depicts the contribution (%) of different food groups to the total nitrite intake among consumers of the same age group. As it shows, processed pork meat was the major contributor for all age groups, with the exception of young adults aged 18–30 years, where poultry meat slightly outperformed (39.1% vs. 38.2% for pork meat), and the elderly (65+ years), where pork meat and sausages were found to contribute equally to total nitrite exposure, at 39.6% and 39.4%, respectively. Moreover, pork meat products were by far the major contributors in children aged 0–9 years (51.9%), with unclassified pork meat contributing the most (25.7%), followed by ham (21.8%). Poultry meat was the second source of nitrite intake in children, with turkey meat accounting for 25.9% of total nitrite intake among children. For adolescents aged 10–17 years, the processed meat that contributed the most to nitrite exposure was pork (39.5%), with ham accounting for 18.1%, but sausages contributed to 30.9% of consumption and poultry meat, primarily turkey (29.5%) also contributed significantly and by about the same level. Young adults aged 18–30 years ingested more nitrite from poultry meat (39.1%, with turkey contributing 37.4%) and pork meat (38.2%), with ham contributing 17.2%, while pork meat was by far the major contributor in adults aged 31–50 years old (40.5%) and in older adults aged 51–64 years (46.8%), followed by poultry meat (35.28%) and sausages (30.94%), respectively. 

## 4. Discussion

The findings of this study indicate that a considerable proportion of Greek consumers (6.6%) may be at risk, as their consumption of nitrite and nitrate (assessed as nitrite equivalent) already exceeded the ADI via ingestion of processed meats only. The risk seems to be higher among children aged 0–9 years, since 15.3% of participants exceeded the ADI, compared to 6.4% in adolescents and 5.7% in adults, a proportion that corresponds to 160.625 children based on the latest data provided by the Hellenic Statistical Authority [43]. The major food groups contributing to total nitrite intake were processed products from meat of pork and turkey, most frequently consumed as part of mixed dishes (mainly in toasts, sandwiches and pizzas).

Due to differences in the design of the studies estimating the nitrate and nitrite intakes from processed meat products that have been conducted at EU level [12] and in other countries [16,17,18,19,20,21,22,23,24] (such as additive evaluations, sources of intake, occurrence data, age groupings, dietary assessment methodologies, conversion factors utilized, and more), the results are quite difficult to compare. Children aged 3 to 9 years in the EU were found to have a mean dietary exposure to nitrites from their use solely as food additives ranging from 0.03 to 0.027 mg/kg bw/day [12]. For this estimate, a sample of 838 children from the Greek island of Crete was included, whose nitrite intake in Tier2 ranged from 0.03 to 0.15, similar to the 0.038 (0.055) mg/kg bw/day found in the present study. The proportion of children exceeding the ADI among children 0–9 years in this study (15.3%) was higher than that of Serbian children < 9 years old (9.33%) [23], Estonian children 1–6 years (3.1%) [24] and Swedish children aged 4, 8–9 and 11–12 years old (0.1%) [44]. Based on the aforementioned studies, mean intakes as % of ADI were 40.0% for Serbian children, 21.9% and 22.9% among Estonian children 12–35 months and 3–10 years, respectively, and 10% for Swedish children, versus the higher 54.3% found for Greek children aged 0–9 years. In comparison to the median of 0.02 mg/kg bw/day and mean of 0.038 mg/kg bw/day for children aged 0–9 years in our study, median nitrite intakes of 0.016, 0.040, and 0.033 mg/kg bw/day were found for children aged 1, 3, and 6 years old in Finland [20] and mean intakes of 0.110–0.521 mg/kg bw/day for children in Sudan [21]. The daily intake of nitrite for adults was estimated to be in the range of 0.027–0.130 mg/kg bw/day in Sudan [21] and 0.038–0.063 mg/kg bw/day for adults and older children aged 4–18 years in the UK [18], in comparison to 0.02 and 0.03 mg/kg bw/day for adults and minors (<18 years) in our study. Finally, the average daily intake of nitrite was 6% of ADI in a Belgian population ≥15 years old (source: processed meat products) [17] and 75.9% for an Austrian adult population (source: meat products and drinking water) [45], compared with 28.6% of Greek adult participants in our study (source: processed meat products). Additionally, preserved meat was found to contribute considerably more to nitrite ingestion in children (81.1%) in our study than in EFSA’s estimate based on the participants from the Greek island of Crete (48.7%), with sausages contributing 17.8% against the 51.3% projected by EFSA. Those differences may be due to the fact that the sample used by EFSA was not representative of the Greek child population. Additionally, the children were aged 3–9 years old, whereas infants and toddlers (0–3 years, N = 15) were also included in the present study. Therefore, the outcomes cannot be compared with precision. Finally, major contributors were minced cooked sausages and canned meat for Serbian children [23] and whole muscle meat cured in brine (54%) and cured, cooked sausages (45%) for the U.S. population ≥2 years [22].

Our study had strengths as well as limitations. As far as we know, this is the first study to use a nationally representative sample to evaluate the nitrite and nitrate intake from processed meat products. The survey-specific FPQs were utilized to adjust for the quantity of processed meat ingested over time. The possible effect of the endogenous conversion of nitrate to nitrite was investigated and the higher 9% conversion factor suggested by EFSA was used [12] in order to securely assess the maximum possible risk for consumers, although the in vivo converted amount is rarely taken into consideration when dietary exposure to nitrite and nitrate through processed foods is studied [20]. As already indicated by experts, in order to achieve consistency and thus more reliable nitrite exposure estimates, the conversion factor has to be defined more accurately [46,47].

Uncertainties in the exposure assessment of nitrite and nitrate intake from processed meat products have been summarized in Table S10, according to the relevant guidance provided by EFSA [48]. The lack of national data on the use and occurrence of nitrite and nitrate salts in processed meat products posed a limitation to this study. The regulatory scenario used is considered the worst case scenario, as it tends to systematically overestimate current intakes [48]; overall, our results could be an overestimation of nitrite ingested from processed meat, and therefore, a more refined assessment may be required. However, the European Commission that suggests using a stepwise procedure to estimate the additive intakes [33], with Tier 3 (based on individual food consumption data and measured data on additives occurrence) to be performed only when Tier 2 is exceeded [49]. Given the difficulty in obtaining accurate concentration data, it has also been suggested to combine the actual national food consumption data with the MPL when the additive is listed in the label of the food product (Tier 2a) [50]. Additionally, EFSA identifies the fact that the compounds could also be used at levels higher than permitted in the legislation as an extrapolation uncertainty affecting the estimation of additives [48]. Furthermore, the reported levels of nitrite and nitrate usage in meat products, as provided by European industry [12,13] were equal to the MPLs, with the exception of E250 when used solely as a flavor enhancer [13]; a detail that could not be taken into account in this study as such detailed information was not provided. 

Some other methodological limitations include the challenge in FoodEx2 codification and mapping with the categories outlined in the legislation [5], as thorough meat product classification may have not been permitted by the information available in the survey. This challenge is also indicated by EFSA, as restrictions/exceptions in the regulation could not be taken under consideration in similar projects [12,35]. Finally, residual levels might be affected by the processing time and temperature, the primary additive dosage, pH, the addition of ascorbate and/or other antioxidant components, and the existence of microorganisms, resulting in a continuous reduction during storage [51]. These factors could not be considered in this risk assessment, as no relevant data were available. 

EFSA concluded that that the ADI would be surpassed at the EU level if all dietary nitrite sources were taken into account at the mean in infants, toddlers and children and at the highest exposure for all age groups [12]. Nitrate intake was also suggested to be exceeded for all age groups at the mean and highest percentiles if all sources of dietary intake (food additives, natural presence and contaminants) were assessed [13]. Other national studies have already evaluated the contribution from the conversion of dietary nitrate to nitrite in various foods, especially vegetables and fruits [19,45], although a direct link between nitrate and nitrite intake from vegetables and fruits and adverse health effects has not been identified [7]. Further research may be restricted by the lack of national data on the concentration of nitrite and nitrate in a number of food categories where these compounds are present as naturally occurring species (vegetable/fruits) or contaminants (drinking water). Surveillance data could allow this uncertainty of over/underestimation to be quantified; therefore, regulatory authorities could plan and maintain a system to monitor the content of nitrite and nitrate in products in which these compounds may occur naturally, as contaminants or additives, within a risk-based approach with appropriate frequency.

Finally, our outcomes are in accordance with the Scientific Committee on Food (SCF) of the European Union that specifically recommended that the subpopulation of children be given special attention, because of their higher ingestion levels relative to their body weight [33]. Increasing public knowledge of the potentially harmful consequences of nitrite and nitrate in processed meats could result in a change in dietary preferences and habits among Greeks, particularly in children and their caretakers. Moreover, the actual nitrite consumption from processed meat products might depend on the type of meat, since chicken products have been suggested to have higher residual nitrite levels than pork and beef products [44]. This could result in an elevated nitrite exposure in consumers switching from consuming processed red meat to white meat products [19] given the public’s perception of poultry meat as being healthier, and could be also considered in future studies and dietary guidelines. If diet habits keep changing, deviating from the Mediterranean diet, and processed meat consumption keeps rising, the intake may increase beyond even higher levels; thus, the idea of the food industry shifting toward using healthier, “greener” preservation technologies is generally supported.

## 5. Conclusions

The median nitrite and nitrate intake from processed meat products, estimated as nitrite equivalent, revealed that a significant proportion of Greek consumers were at risk of exceeding the ADI for nitrite from the consumption of processed meat alone, mainly that of processed products from pork and turkey meat consumed as part of mixed dishes (more frequently on toast, sandwiches, and pizza). Special attention should be given to children aged 0–9 years, who had by far the highest proportion of exceeders among them. Considering the cumulative impact of chronic exposure to additional dietary sources of nitrite and nitrate, these results are alarmingly high and could indicate the need for competent authorities to establish relevant educational campaigns aiming to raise public awareness of the potential adverse health effects of nitrite and nitrate found in processed meat. Public awareness creates indirect pressure on the food industry and could effectively lead to the decrease in or even elimination of these compounds. Other techniques that will adequately support food safety may be developed to replace these additives. Furthermore, competent authorities should develop and maintain a monitoring plan for the nitrite and nitrate content of various products in the Greek market. Future research could assess the nitrite and nitrate dietary intakes using refined occurrence data. Finally, potential associations of dietary intakes of nitrite and nitrate with adverse health effects other than cancer, in total but especially for those at risk, could be explored if relevant data are made available.

## Figures and Tables

**Table 1:** Summary of dietary exposure to nitrite from processed meat products (in mg/kg bw/day and as % of ADI).

Age Group	Dietary Exposure to Nitrite					
	Median		Mean		95th Percentile	
	mg/kg bw/day	% ADI ^1^	mg/kg bw/day	% ADI	mg/kg bw/day	% ADI
Minors (<18 years) ^2^	0.014	14.3	0.030	42.9	0.126	180.0
Children 0–9 years	0.02	28.6	0.038	54.3	0.173	247.1
Adolescents 10–17 years	0.01	14.3	0.022	31.4	0.076	108.6
Adults (≥18 years) ^3^	0.007	10.0	0.021	30.0	0.078	111.4
Young adults 18–30 years	0.008	11.4	0.025	35.7	0.087	124.3
Adults 31–50 years	0.007	10	0.020	28.6	0.079	112.9
Older adults 51–64 years	0.004	5.7	0.014	20.0	0.056	80
The elderly ≥65 years	0.003	4.3	0.008	11.4	0.048	68.6
Total	0.007	10	0.022	31.4	0.173	118.6

^1^ ADI: Acceptable Daily Intake.

**Table 2:** Baseline variables of the participants by level of intake (above and below ADI of 0.07 mg/kg bw/day).

Variable ^1^	Total N = 2152	Below ADI N = 2009 (93.4%)	Above ADI N = 143 (6.6%)	*p*-Value (by Intake Level) ^2^
Age(years), median (IQR)	29 (21, 42)	29 (21, 42)	26 (15, 36)	<0.001
Age group (years), *n* (%)				<0.001
Children (0–9)	190 (8.8)	161 (8.0)	29 (20.3)	
Adolescents (10–17)	188 (8.7)	176 (8.8)	12 (8.4)	
Young adults (18–30)	782 (36.4)	729 (36.3)	53 (37.0)	
Adults (31–50)	664 (30.9)	624 (31.0)	40 (28.0)	
Older adults (51–64)	211 (9.8)	203 (10.1)	8 (5.6)	
Elderly (>=65)	117 (5.4)	116 (5.8)	1 (0.7)	
Sex, *n* (%)				<0.001
Females	1144 (53.3)	1088 (54.4)	56 (39.2)	
Males	1001 (46.7)	914 (45.6)	87 (60.8)	
Weight (kg), mean (sd)	67.9 (20.7)	68.2 (20.4)	62.9 (24.2)	<0.05
Area of residence, *n* (%)				0.215
Attiki and Thessaloniki	1429 (67.0)	1335 (67.1)	94 (66.7)	
Islands (including Crete)	219 (10.3)	199 (10.0)	20 (14.2)	
Mainland	483 (22.7)	456 (22.9)	27 (19.1)	
Marital status ^3^, *n* (%)				0.165
Divorced/Separated/Widowed/Single	1044 (62.0)	980 (61.6)	64 (68.8)	
Married/Cohabiting	639 (38.0)	610 (38.4)	29 (31.2)	
Education level ^3^, *n* (%)				0.146
Up to 6 years of school	88 (5.1)	87 (5.3)	1 (1.1)	
12 years of school	661 (38.4)	626 (38.5)	35 (36.8)	
Higher education (including colleges)	974 (56.5)	915 (56.2)	59 (62.1)	
Employment status ^3^, *n* (%)				0.034
Unemployed	535 (31.0)	507 (31.1)	28 (29.2)	
Employed	1009 (58.5)	944 (58.0)	65 (67.7)	
Pension	180 (10.5)	177 (10.9)	3 (3.1)	
Smoking status ^3^, *n* (%)				0.119
Never smoker	847 (48.1)	801 (48.3)	46 (45.1)	
Current smoker	656 (37.3)	622 (37.5)	34 (33.3)	
Ex-smoker	257 (14.6)	235 (14.2)	22 (21.6)	
Physical activity status ^3,4^, *n* (%)				0.930
Low	255 (15.5)	239 (15.3)	16 (17.2)	
Moderate	624 (37.8)	588 (37.8)	36 (38.7)	
Sedentary	101 (6.1)	95 (6.1)	6 (6.5)	
Very	670 (40.6)	635 (40.8)	35 (37.6)	
Total energy intake (kcal/day), median (IQR)	1917.8 (1443.1, 2518.5)	1894.4 (1433.7, 2493.3)	2249.9 (1639.6, 3357.4)	<0.001
Total fat intake (%energy) ^3^, mean (sd)	38.1 (9.5)	38.0 (9.5)	39.6 (9.5)	0.1246
Total TFA intake (%energy) ^3^, median (IQR)	0.6 (0.4, 0.8)	0.5 (0.4, 0.8)	0.6 (0.4, 0.8)	0.3107
Total SFA intake (%energy) ^3^, mean (sd)	13.4 (4.0)	13.4 (4.0)	13.9 (4.0)	0.2235
Total sodium intake (mg), mean (sd)	2303 (690.1)	2276.3 (666.2)	2770.3 (906.1)	<0.001
Sodium intake ^3^, *n* (%)				<0.001
<1500	104 (6.4)	103 (6.7)	1 (1.1)	
>=1500 and <2300	851 (52.3)	816 (53.1)	35 (38.5)	
>=2300	673 (41.3)	618 (40.2)	55 (60.4)	
BMI adults(kg/m^2^) ^3,5^ mean (sd)	24.0 (5.2)	24.0 (5.2)	23.0 (5.1)	0.020
BMI adults categories ^3,5^, *n* (%)				0.488
Healthy weight	977 (56.6)	921 (56.6)	56 (57.1)	
Overweight	507 (29.4)	475 (29.2)	32 (32.7)	
Obese	241 (14.0)	231 (14.2)	10 (10.2)	
BMI children ^6^, mean (sd)	18.8 (4.2)	18.9 (4.3)	18.1 (3.8)	0.2680
BMI children categories ^5,6^, *n* (%)				0.602
Healthy weight	332 (92.2)	293 (91.8)	39 (95.1)	
Overweight	21 (5.8)	19 (6.0)	2 (4.9)	
Obese	7 (2.0)	7 (2.2)	0 (0)	
MedDiet score ^3^, mean (sd)	27.0 (6.4)	27.1 (6.3)	25.0 (6.5)	<0.05
MedDiet category ^3^, *n* (%)				<0.05
MD < 23	373 (22.9)	341 (22.2)	32 (35.2)	
MD >= 23	1255 (77.1)	1196 (77.8)	59 (64.8)	

^1^ Continuous variables were presented as mean and standard deviation (sd) when normally distributed and median and interquartile range (IQR: 25th percentile, 75th percentile) when skewed. Categorical variables were presented as frequencies. ^2^ Group differences were tested using chi square test for proportions and Kruskall–Wallis rank sum or ANOVA test depending on data distribution. Level of significance was set at alpha = 5%. ^3^ Adults only. ^4^ Physical activity (PA) was defined according to the International Physical Activity Questionnaire (IPAQ). ^5^ Body Mass Index (BMI) was calculated from measurements of weight (kg) and height (m): weight/height^2^ (kg/m^2^). Weight status was categorized as healthy weight ≤25 kg/m, 25 ≤ overweight < 30 kg/m^2^, and obese ≥ 30 kg/m^2^. Children and adolescents were classified using the extended International Obesity Task Force (IOTF) tables [39]. ^6^ Children only.

## Data Availability

Raw data were generated at the Agricultural University of Athens. Data supporting the findings of this study are available from the corresponding author on request.

## Supplementary Materials

The following supporting information can be downloaded at: https://www.mdpi.com/article/10.3390/ijerph191912800/s1, Table S1: Food groups and items (including composite disks and recipes) involved in the exposure assessment; Table S2: Frequency of processed meat products and mixed dishes/recipes containing processed meat products in HNNHS; Table S3: Concentrations levels of nitrite (E 249–250) and nitrate (E 251–252) used in the regulatory maximum level scenario (mg/kg); Table S4: Dietary daily intakes of nitrate, nitrite and combined nitrite and nitrate via processed meat products using different nitrate-to-nitrite conversion factors (a) for general population and (b) for consumers only; Table S5: Distribution of daily nitrite intake (in mg/kg bw/day and as % of ADI), estimated in total and per sex and age group for consumers only; Table S6: Likelihood of exceeding ADI of nitrite from total nitrite intake from processed meat products; Table S7: Proportion of population per frequency of consumption of processed meat products, as reported in HNNHS, among (a) consumers and (b) consumers with total nitrite intake exceeding the ADI of 0.07 mg/kw bw/day; Table S8: Consumers with total nitrite intake from processed meat products above the ADI of nitrite (0.07 mg/kg bw/day) per sex and age group in number of individuals (*n*), as a percentage of individuals of the same age group [a = (*n*/N) × 100] and as a percentage of total number of individuals exceeding ADI (*n* = 143) [b = (*n*/143)*100]; Figure S1: Main FoodEx2 group contribution to total nitrite intake among processed meat products; Table S9: Contribution (%) of meat type and FoodEx2 food groups to total nitrite exposure per age group; Table S10. Qualitative evaluation of influence of uncertainties on the dietary exposure estimated.
