# Supplementary material for: Risk Assessment of Nitrite and Nitrate Intake from Processed Meat Products: Results from the Hellenic National Nutrition and Health Survey (HNNHS)
Source: Int J Environ Res Public Health. 2022 Oct 6;19(19):12800. doi: 10.3390/ijerph191912800 (PMC9565037; doi:10.3390/ijerph191912800)
Supplement: Supplementary file 1 [file ijerph-19-12800-s001.zip › ijerph-1944905-Supplementary.pdf]

Table S1. Food groups and items (including composite disks and recipes) involved in the exposure assessment.

**FOODEX Category**
**Food group (if different than FOODEX Category)**
**Food subgroup (if any)**
**x. FoodEx2 Code & Term**
HNNHS Foods
**PRESERVED MEAT**
**Pork Meat**
**Pork meat, Bacon**
**1. A022X Bacon**
Caesar salad
Cheese soufflé
Processed meat products, bacon
Crepes
Hamburger
Lasagna soufflé
Omelet
Pasta au gratin
Pasta carbonara
Peinirli ("pizza boat")
Pizza
Potatoes au gratin
Sandwich
Soup
Tart
Toast
**Pork meat, Ham**
**2. A022T Ham, pork**
Caesar salad
Cheese soufflé
Chef salad
Chicken with ham
Processed meat products, ham
Crepes
Croissant
Lasagna soufflé
Omelet
Pasta au gratin
Peinirli ("pizza boat")
Pie
Pizza
Sandwich
Toast
Tortilla sandwich
**3. A023K Cooked pork ham**
Caesar salad

FOODEX Category<br>Food group (if different than FOODEX Category)<br>Food subgroup (if any)<br>x. FoodEx2 Code & Term<br>HNNHS Foods
Chef salad
Processed meat products, ham
Crepes
Lasagna soufflé
Peinirli ("pizza boat")
Pie
Pizza
Sandwich
**Pork meat, other**
**4. A022S Cured seasoned pork meat**
Caesar salad
Chef salad
Crepes
Pasta au gratin
Pie
Pizza
Sandwich
Processed meat products
Processed meat products, prosciutto
Toast
Meat preserved with salt, "apaki"
Meat preserved with salt, pork
**5. A023H Cooked cured (or seasoned) pork meat**
Processed meat products
Toast
Tortilla sandwich
Pizza
Sandwich
Processed meat products, prosciutto
Crepes
**Poultry meat**
**Poultry meat, Chicken**
**6. A023S Cooked cured (or seasoned) poultry meat**
Processed meat products, chicken
Sandwich
Toast
**7. A023X Cooked other poultry meat**
Processed meat products, chicken
**Poultry meat, Turkey**
**8. A023E Cured seasoned poultry meat**
Processed meat products, turkey
Pasta carbonara

FOODEX Category<br>Food group (if different than FOODEX Category)<br>**Food subgroup (if any)**<br>**x. FoodEx2 Code & Term**<br>HNNHS Foods
Sandwich
Toast
**9. A023T Cooked turkey meat**
Processed meat products, turkey
Crepes
Croissant
Omelet
Pasta carbonara
Pizza
Salad
Sandwich
Toast
Tomato sauce
Tortilla sandwich
SAUSAGES
**10. A0EYP Preserved or partly preserved sausages**
Beans
Peinirli (“pizza boat”)
Pie
Piroshky
Pizza
Sandwich
Sausage
Sausage, breakfast type
“Spetsofai” (Sausages with peppers)
Tomato sauce
Sausage, soutzouki
Sausage, turkey
Sausage, tzoumagias-type
Sausage, village type
Toast
Sausage, cocktail-type
**11. A025C Chorizo and similar**
Paella
**12. A026B Frankfurt-type sausage**
Hot dog
Sandwich
Sausage, Frankfurt
Tomato sauce
**13. A025Q Mortadella-type sausage**
Processed meat products, mortadella
Sandwich
Toast

FOODEX Category
Food group (if different than FOODEX Category)
Food subgroup (if any)
x. FoodEx2 Code & Term
HNNHS Foods

14. A025B Pepperoni/paprika-type sausage
Pizza

15. A024X Salami-type sausage
Processed meat products, air-dried salami
Processed meat products, Hellenic-type salami
Processed meat products, salami
Processed meat products, salami Hungarian type
Omelet
Pizza
Sandwich
Sausage, beer salami
Sausage, salami
Souvlaki
Toast

MEAT SPECIALTIES

16. A026M Liver based spreadable-textured specialties
Pate and meat pastes, pate, duck liver
Pate and meat pastes, pate, other poultry liver

17. A026Q Pate, chicken liver
Pate and meat pastes, pate, chicken liver

Table S2. Frequency of processed meat products and mixed dishes/recipes containing processed meat products in HNNHS.

Foods/Mixed Dishes/Recipes	Frequency in HNNHS (%)
**Foods**	**24.91%**
Processed meat products	1.85%
Processed meat products, turkey	10.92%
Processed meat products, air-dried salami	0.87%
Processed meat products, bacon	0.34%
Processed meat products, chicken	1.08%
Processed meat products, ham	4.35%
Processed meat products, Hellenic salami	0.08%
Processed meat products, loin	0.44%
Processed meat products, mortadella	0.36%
Processed meat products, prosciutto	0.20%
Processed meat products, salami	0.03%
Processed meat products, salami Hungarian type	0.08%
Pate and meat pastes, pate, chicken liver	0.08%
Pate and meat pastes, pate, duck liver	0.03%
Pate and meat pastes, pate, other poultry liver	0.06%
Meat preserved with salt, "apaki"	0.11%
Meat preserved with salt, pork	0.06%
Sausages	1.65%
Sausage, beer salami	0.06%
Sausage, breakfast type	0.03%
Sausage, cocktail	0.08%
Sausage, Frankfurt	0.08%
Sausage, salami	0.08%
Sausage, soutzouki	0.14%
Sausage, turkey	0.14%
Sausage, tzoumagias-type	0.59%
Sausage, village type	1.12%
**Mixed dishes/Recipes**	**75.09%**
Beans	0.03%
Caesar salad	0.47%
Cheese soufflé	0.06%
Chef salad	0.28%
Chicken with ham	0.56%
Crepes	1.43%
Croissant	0.64%
Hamburger	0.59%
Hot dog	0.73%
Lasagna soufflé	1.42%
Omelet	0.36%
Paella	0.42%
Pasta au gratin	1.59%
Pasta carbonara	1.71%

Foods/Mixed Dishes/Recipes	Frequency in HNNHS (%)
Peinirli ("pizza boat")	1.01%
Pie	4.13%
Piroshky	0.45%
Pizza	14.24%
Potatoes au gratin	0.17%
Salad	0.03%
Sandwich	7.58%
Soup	0.03%
"Spetsofai" (Sausages with peppers)	0.36%
Tart	0.03%
Toast	36.61%
Tomato sauce	0.12%
Tortilla sandwich	0.03%
Souvlaki	0.03%
**Total**	**100.00%**

Table S3. Concentration levels of nitrite (E 249-250) and nitrate (E 251-252) used in the regulatory maximum level scenario (mg/kg).

Food category number	Food category name	FOODEX category	FOODEX Code	FOODEX Name	Frequency in HNNHS %	NITRITE MPL (mgkg)	NITRATE MPL (mgkg)
08.3.1	Non-heat-treated meat products	Preserved meat	A022S	Cured seasoned pork meat	1.08	150	150
08.3.1	Non-heat-treated meat products	Preserved meat	A022X	Bacon	9.02	150	150
08.3.1	Non-heat-treated meat products	Preserved meat	A023E	Cured seasoned poultry meat	3.26	150	150
08.3.1	Non-heat-treated meat products	Sausages	A024X	Salami-type sausage	1.77	150	150
08.3.1	Non-heat-treated meat products	Sausages	A025B	Pepperoni/paprika-type sausage	0.63	150	150
08.3.1	Non-heat-treated meat products	Sausages	A025C	Chorizo and similar	0.41	150	150
**08.3.1**	**Total**				**16.17**		
08.3.2	Heat–treated meat products	Preserved meat	A022T	Ham, pork	23.97	150	
08.3.2	Heat–treated meat products	Preserved meat	A023H	Cooked cured (or seasoned) pork meat	21.13	150	
08.3.2	Heat–treated meat products	Preserved meat	A023K	Cooked pork ham	2.43	150	
08.3.2	Heat–treated meat products	Preserved meat	A023S	Cooked cured (or seasoned) poultry meat	1.38	150	
08.3.2	Heat–treated meat products	Preserved meat	A023T	Cooked turkey meat	25.71	150	
08.3.2	Heat–treated meat products	Preserved meat	A023X	Cooked other poultry meat	0.08	150	
08.3.2	Heat–treated meat products	Sausages	A025Q	Mortadella-type sausage	0.63	150	
08.3.2	Heat–treated meat products	Sausages	A026B	Frankfurt-type sausage	0.86	150	
08.3.2	Heat–treated meat products	Meat specialties	A026Q	Pate, chicken liver	0.08	150	
08.3.2	Heat–treated meat products	Meat specialties	A026M	Liver based spreadable-textured specialties	0.08	150	
08.3.2	Heat–treated meat products	Sausages	A0EYP	Preserved or partly preserved sausages	7.48	150	
**08.3.2**	**Total**				**83.83**		
**TOTAL**					**100.00**		

Table S4. Dietary daily intakes of nitrate, nitrite and combined nitrite and nitrate via processed meat products using different nitrate-to-nitrite conversion factors (a) for general population and (b) for consumers only.

Population	Intakes	Median (IQR)	p90	p95
**General population (N=4532)**	Nitrate (mg/kg bw/day)	0 (0, 0)	0	0.003
	Nitrite (mg/kg bw/day)	0 (0, 0.007)	0.023	0.048
	Nitrite and nitrate, with nitrate-to-nitrite conversion factor of 1% (mg/kg bw/day)	0 (0, 0.007))	0.023	0.048
	Nitrite and nitrate, with nitrate-to-nitrite conversion factor of 2.3% (mg/kg bw/day)	0 (0, 0.007))	0.024	0.048
	Nitrite and nitrate, with nitrate-to-nitrite conversion factor of 9% (mg/kg bw/day)	0 (0, 0.007)	0.023	0.049
	Nitrite and nitrate intake[1] (% ADI[2])	0 (0, 10.0)	32.9	70.0
**Consumers only (N=2152, 47.5% of the general population)**	Nitrate (mg/kg bw/day)	0 (0, 0)	0.003	0.012
	Nitrite (mg/kg bw/day)	0.007 (0.003, 0.020)	0.050	0.082
	Nitrite and nitrate, with nitrate-to-nitrite conversion factor of 1% (mg/kg bw/day)	0.007 (0.003, 0.020)	0.050	0.082
	Nitrite and nitrate, with nitrate-to-nitrite conversion factor of 2.3% (mg/kg bw/day)	0.007 (0.003, 0.020)	0.050	0.083
	Nitrite and nitrate, with nitrate-to-nitrite conversion factor of 9% (mg/kg bw/day)	0.007 (0.003, 0.020)	0.051	0.083
	Nitrite and nitrate intake[1] (% ADI[2])	10.0 (4.3, 28.6)	72.9	118.6

1. Using nitrate-to-nitrite conversion factor of 9%
2. ADI of 0.07mg/kg bw/day for nitrite

Tabel S5. Distribution of daily nitrite intake (in mg/kg bw/day and as %of ADI), estimated in total and per sex and age group for consumers only.

Age group (years)	N	%	Daily nitrite intake (mg/kg bw/day								Contribution to ADI (%)							
			p25	p50	p75	p90	p95	p99[1]	Mean	SD	p25	p50	p75	p90	p95	p99[1]	Mean	SD
**FEMALES**	**1144**	**53.3**	**0.003**	**0.007**	**0.018**	**0.044**	**0.067**	**0.309**	**0.022**	**0.064**	**4.3**	**10.0**	**25.7**	**62.9**	**95.7**	**441.4**	**31.4**	**91.4**
**Minors (<18)**	**170**	**14.8**	**0.005**	**0.014**	**0.032**	**0.062**	**0.134**	0.359	**0.032**	**0.065**	**7.1**	**20.0**	**45.7**	**88.6**	**191.4**	512.9	**45.7**	**92.9**
Children (0-9)	85	7.4	0.006	0.019	0.034	0.081	0.161	0.359	0.037	0.059	8.6	27.1	48.6	115.7	230.0	512.9	52.9	84.3
Adolescents (10-17)	85	7.4	0.004	0.011	0.024	0.051	0.076	0.608	0.027	0.070	5.7	15.7	34.3	72.9	108.6	868.6	38.6	100.0
**Adults (≥18)**	**974**	**85.2**	**0.003**	**0.007**	**0.015**	**0.038**	**0.064**	**0.309**	**0.020**	**0.064**	**4.3**	**10.0**	**21.4**	**54.3**	**91.4**	**441.4**	**28.6**	**91.4**
Young adults (18-30)	442	38.7	0.003	0.008	0.019	0.048	0.077	0.332	0.025	0.081	4.3	11.4	27.1	68.6	110.0	474.3	35.7	115.7
Adults (31-50)	348	30.4	0.003	0.007	0.015	0.034	0.057	0.181	0.017	0.042	4.3	10.0	21.4	48.6	81.4	258.6	24.3	60.0
Older adults (51-64)	129	11.3	0.002	0.004	0.012	0.028	0.045	0.076	0.014	0.054	2.9	5.7	17.1	40.0	64.3	108.6	20.0	77.1
Elderly (≥65)	55	4.8	0.002	0.004	0.011	0.038	0.067	0.073	0.011	0.018	2.9	5.7	15.7	54.3	95.7	104.3	15.7	25.7
**MALES**	**1001**	**46.7**	**0.003**	**0.007**	**0.022**	**0.058**	**0.098**	**0.220**	**0.023**	**0.049**	**4.3**	**10.0**	**31.4**	**82.9**	**140.0**	**314.3**	**32.9**	**70.0**
**Minors (<18)**	**206**	**20.6**	**0.006**	**0.014**	**0.030**	**0.075**	**0.126**	0.195	**0.029**	**0.042**	**8.6**	**20.0**	**42.9**	**107.1**	**180.0**	278.6	**41.4**	**60.0**
Children (0-9)	105	10.5	0.009	0.020	0.046	0.115	0.177	0.195	0.040	0.051	12.9	28.6	65.7	164.3	252.9	278.6	57.1	72.9
Adolescents (10-17)	101	10.1	0.003	0.010	0.023	0.048	0.072	0.106	0.019	0.026	4.3	14.3	32.9	68.6	102.9	151.4	27.1	37.1
**Adults (≥18)**	**795**	**79.4**	**0.003**	**0.006**	**0.019**	**0.054**	**0.090**	**0.296**	**0.022**	**0.051**	**4.3**	**8.6**	**27.1**	**77.1**	**128.6**	**422.9**	**31.4**	**72.9**
Young adults (18-30)	338	33.8	0.003	0.008	0.023	0.058	0.097	0.319	0.025	0.056	4.3	11.4	32.9	82.9	138.6	455.7	35.7	80.0
Adults (31-50)	313	31.3	0.003	0.007	0.019	0.061	0.099	0.220	0.023	0.054	4.3	10.0	27.1	87.1	141.4	314.3	32.9	77.1
Older adults (51-64)	82	8.2	0.002	0.004	0.007	0.042	0.084	0.140	0.013	0.027	2.9	5.7	10.0	60.0	120.0	200.0	18.6	38.6
Elderly (>=65)	62	6.1	0.001	0.002	0.006	0.012	0.014	0.049	0.005	0.009	1.4	2.9	8.6	17.1	20.0	70.0	7.1	12.9
**TOTAL**	**2152**	**100**	**0.003**	**0.007**	**0.020**	**0.051**	**0.083**	**0.266**	**0.022**	**0.058**	**4.3**	**10.0**	**28.6**	**72.9**	**118.6**	**380.0**	**31.4**	**82.9**
**Minors (<18)**	**378**	**17.5**	**0.005**	**0.014**	**0.030**	**0.072**	**0.126**	**0.241**	**0.030**	**0.053**	**7.1**	**20.0**	**42.9**	**102.9**	**180.0**	**344.3**	**42.9**	**75.7**
Children (0-9)	190	8.8	0.008	0.02	0.042	0.102	0.173	0.269	0.038	0.055	11.4	28.6	60.0	145.7	247.1	384.3	54.3	78.6
Adolescents (10-17)	188	8.7	0.004	0.01	0.023	0.049	0.076	0.19	0.022	0.051	5.7	14.3	32.9	70.0	108.6	271.4	31.4	72.9
**Adults (≥18)**	**1774**	**82.5**	**0.003**	**0.007**	**0.017**	**0.046**	**0.078**	**0.296**	**0.021**	**0.058**	**4.3**	**10.0**	**24.3**	**65.7**	**111.4**	**422.9**	**30.0**	**82.9**
Young adults (18-30)	782	36.3	0.003	0.008	0.022	0.052	0.087	0.332	0.025	0.071	4.3	11.4	31.4	74.3	124.3	474.3	35.7	101.4
Adults (31-50)	664	30.9	0.003	0.007	0.017	0.041	0.079	0.22	0.02	0.048	4.3	10.0	24.3	58.6	112.9	314.3	28.6	68.6
Older adults (51-64)	211	9.8	0.002	0.004	0.01	0.03	0.056	0.113	0.014	0.045	2.9	5.7	14.3	42.9	80.0	161.4	20.0	64.3
Elderly (>=65)	117	5.5	0.001	0.003	0.007	0.013	0.048	0.067	0.008	0.014	1.4	4.3	10.0	18.6	68.6	95.7	11.4	20.0

1. Calculations at the 99th percentiles when the number of subjects is lower than 300 (children, adolescents, older adults and elderly) have been indicated with grey colour, as those results may not be statistically robust and should be interpreted cautiously [1]

Table S6. Likelihood of exceeding ADI of nitrite from total nitrite intake from processed meat products.

Level of nitrite intake	Odds Ratio	Std. Err.	z	P>z	[95% Conf.	Interval]
Age group						
31-50	0.777991	0.194701	-1	0.316	0.476379	1.270566
51-64	0.6998	0.317109	-0.79	0.431	0.287911	1.700942
65+	0.238563	0.285731	-1.2	0.231	0.022809	2.495178
Sex	1.285699	0.370155	0.87	0.383	0.731268	2.260485
Weight	0.999971	0.008643	0	0.997	0.983173	1.017056
Employment status						
Pension	0.725481	0.553252	-0.42	0.674	0.162742	3.234091
Unemployed	0.894391	0.231454	-0.43	0.666	0.538579	1.485268
Total energy intake	1.000468	0.000113	4.14	0	1.000246	1.000689
Sodium intake category						
>=1500 & <2300	2.672544	2.743197	0.96	0.338	0.357454	19.98156
>=2300	3.117198	3.242189	1.09	0.274	0.405912	23.93849
Med diet category						
MD>=23	0.567615	0.134508	-2.39	0.017	0.356734	0.903157

Table S7. Proportion of population per frequency of consumption of processed meat products, as reported in HNNHS, among (a) consumers and (b) consumers with total nitrite intake exceeding the ADI of 0.07mg/kw bw/day.

Frequency of consumption of processed meat products	(a) Consumers (%)	(b) Consumers with total nitrite intake ≥ADI (%)
Less than once a month	3.0	0
1 to 3 times per month	22.95	0
once a week	54.32	6.29
2-4 times a week	17.34	39.16
5-6 times a week	1.65	16.78
every day	0.48	19.58
2 to 3 times a day	0.20	12.59
4 to 5 times a day	0.05	4.90
5 to 6 times a day	0.01	0.70
Total	100.00	100.00

Table S8. Consumers with total nitrite intake from processed meat products above the ADI of nitrite (0.07mg/kg bw/day) per sex and age group in number of individuals (n), as a percentage of individuals of the same age group [a=(n/N) × 100] and as a percentage of total number of individuals exceeding ADI (n=143) [b=(n/143)) × 100].

**Age group**	**N**	**Consumers with nitrite intake above ADI**		
		**n**	**(a)** % of consumers within the same sex/age group	**(b)** % of total consumers exceeding ADI
**Females**	**1144**	**56**	**4.9**	**39.2**
Children (0-9y)	85	10	11.8	7.0
Adolescents (10-17y)	85	5	5.9	3.5
Young adults (18-30y)	442	26	5.9	18.2
Adults (31-50y)	348	12	3.4	8.4
Older adults (51-64y)	129	2	1.6	1.4
Elderly (>=65y)	55	1	1.8	0.7
**Males**	**1001**	**87**	**8.7**	**60.8**
Children (0-9y)	105	19	18.1	13.3
Adolescents (10-17y)	101	7	6.9	4.9
Young adults (18-30y)	338	27	8.0	18.9
Adults (31-50y)	313	28	8.9	19.6
Older adults (51-64y)	82	6	7.3	4.2
Elderly (>=65y)	62	0	0.0	0.0
**Total**	**2152**	**143**	**6.6**	**100.0**
**<18 years**	**378**	**41**	**10.9**	**28.7**
Children (0-9y)	190	29	15.3	20.3
Adolescents (10-17y)	188	12	6.4	8.4
**>=18 years**	**1774**	**102**	**5.7**	**71.3**
Young adults (18-30y)	782	53	6.8	37.1
Adults (31-50y)	664	40	6.0	28.0
Older adults (51-64y)	211	8	3.8	5.6
Elderly (>=65y)	117	1	0.9	0.7

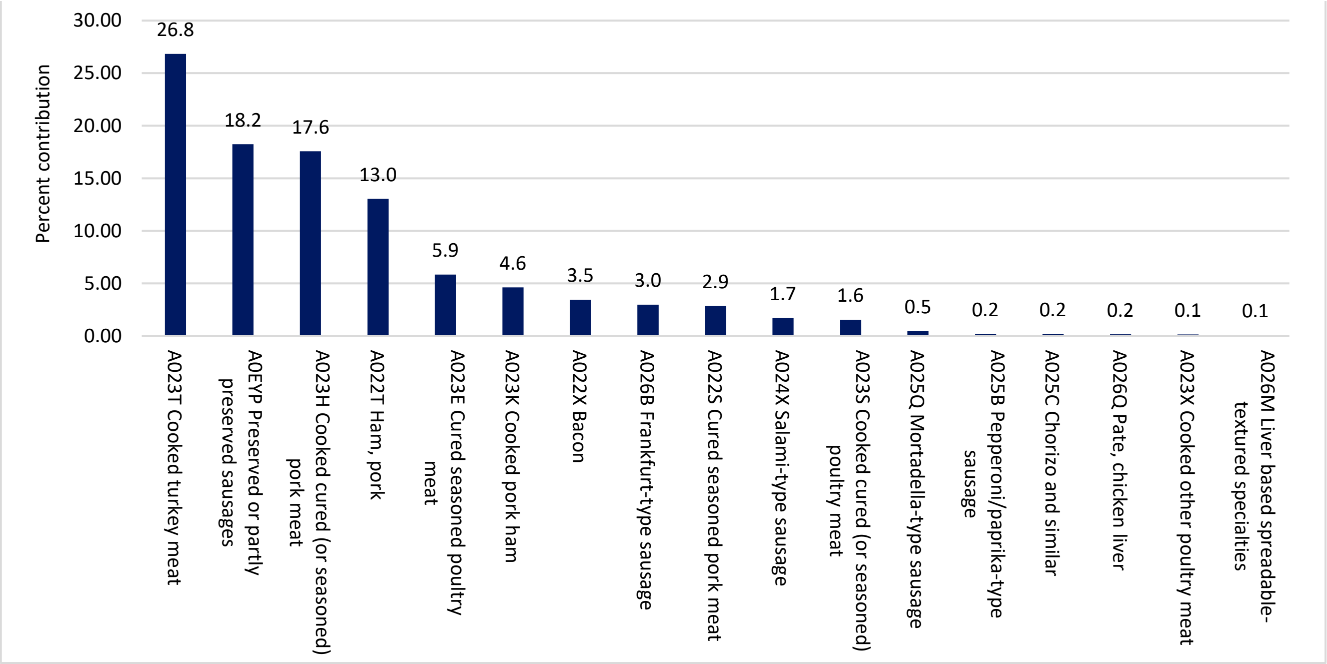

Figure S1. Main FoodEx2 group contribution (%) to total nitrite intakeamong processed meat products.

Table S9. Contribution (%) of meat type and FoodEx2 food groups to total nitrite exposure per age group.

FOODEX CATEGORY<br>Food Group (if different than FOODEX Category)<br>Food Subgroup (if any)<br>FoodEx2 Category	Age group (years)						TOTAL
	0-9	10-17	18-30	31-50	51-64	65+	
**PRESERVED MEAT**							
**Pork meat**	**51.91**	**39.05**	**38.23**	**40.45**	**46.77**	**39.57**	**41.53**
**Pork meat, ham**	**21.84**	**18.01**	**17.19**	**14.82**	**21.47**	**21.43**	**17.66**
A022T Ham, pork	17.98	14.72	11.05	12.26	12.53	20.90	13.04
A023K Cooked pork ham	3.86	3.29	6.14	2.55	8.94	0.53	4.62
**Pork meat, bacon**	**4.40**	**3.43**	**3.32**	**3.60**	**2.34**	**0.63**	**3.46**
A022X Bacon	4.40	3.43	3.32	3.60	2.34	0.63	3.46
**Pork meat, other**	**25.67**	**17.61**	**17.72**	**22.04**	**22.95**	**17.51**	**20.41**
A023H Cooked cured (or seasoned) pork meat	23.65	15.91	14.37	19.25	18.71	16.72	17.56
A022S Cured seasoned pork meat	2.02	1.70	3.35	2.79	4.24	0.79	2.85
**Poultry meat**	**29.23**	**30.07**	**39.08**	**35.28**	**22.27**	**21.07**	**34.39**
**Poultry meat, turkey**	**25.87**	**29.54**	**37.35**	**34.24**	**19.99**	**20.01**	**32.68**
A023T Cooked turkey meat	24.99	27.88	28.21	29.23	12.70	17.80	26.83
A023E Cured seasoned poultry meat	0.88	1.66	9.14	5.01	7.29	2.21	5.85
**Poultry meat, other (chicken)**	**3.36**	**0.53**	**1.73**	**1.04**	**2.27**	**1.06**	**1.71**
A023S Cooked cured (or seasoned) poultry meat	2.55	0.53	1.70	0.99	2.27	1.06	1.56
A023X Cooked other poultry meat	0.82	0.00	0.03	0.05	0.00	0.00	0.15
**SAUSAGES**	**17.82**	**30.88**	**22.68**	**23.92**	**30.94**	**39.36**	**23.82**
A024X Salami-type sausage	3.96	0.39	0.95	2.11	1.95	0.16	1.72
A025B Pepperoni/paprika-type sausage	0.71	0.00	0.12	0.14	0.31	0.17	0.22
A025C Chorizo and similar	0.00	0.32	0.35	0.01	0.03	0.02	0.18
A025Q Mortadella-type sausage	0.00	0.00	0.36	0.26	4.01	1.96	0.50
A026B Frankfurt-type sausage	1.88	0.82	4.12	3.42	0.18	0.05	2.98
A0EYP Preserved or partly preserved sausages	11.26	29.35	16.78	17.99	24.46	37.00	18.23
**MEAT SPECIALTIES**	**1.04**	**0.00**	**0.01**	**0.35**	**0.02**	**0.00**	**0.26**
A026M Liver based spreadable-textured specialties	0.00	0.00	0.01	0.35	0.02	0.00	0.10
A026Q Pate, chicken liver	1.04	0.00	0.00	0.00	0.00	0.00	0.16

Table S10. Qualitative evaluation of influence of uncertainties on the dietary exposure estimated**.**

Sources of uncertainties		Direction
1.	Regulatory maximum level exposure assessment scenario (food categories authorized at MPL according to Annex II to Regulation (EC) No 1333/2008	+
2.	Extrapolation from recipes to their ingredients.	±
3.	Uncertainty of nitrate-to-nitrite conversion factor	±
4.	Misclassification of meat products; incorrect FoodEx coding and mismatch with food categories of Regulation (EC) 1333/2008	±
5.	No use of procession factors	±

+, uncertainty with potential to cause overestimation of exposure

-, uncertainty with potential to cause underestimation of exposure

Reference

1. EFSA Use of the EFSA Comprehensive European Food Consumption Database in Exposure Assessment. *EFSA J.* **2011**, *9*, doi:10.2903/j.efsa.2011.2097.
